# Health Implications of Widespread Micro- and Nanoplastic Exposure: Environmental Prevalence, Mechanisms, and Biological Impact on Humans

**DOI:** 10.3390/toxics12100730

**Published:** 2024-10-10

**Authors:** Olivia-Teodora Preda, Ana-Maria Vlasceanu, Cristina Veronica Andreescu, Aristidis Tsatsakis, Yaroslav Mezhuev, Carolina Negrei, Daniela Luiza Baconi

**Affiliations:** 1 Department of Toxicology, Carol Davila University of Medicine and Pharmacy, 37 Dionisie Lupu Street, Sector 2, 20021 Bucharest, Romania; olivia-teodora.preda@drd.umfcd.ro (O.-T.P.); daniela.baconi@umfcd.ro (D.L.B.); 2 Department of Foreign Languages, Carol Davila University of Medicine and Pharmacy, 37 Dionisie Lupu Street, Sector 2, 20021 Bucharest, Romania; cristina.andreescu@umfcd.ro; 3 Center of Toxicology Science & Research, Division of Morphology, Medical School, University of Crete, Voutes Campus, 71003 Heraklion, Greece; tsatsaka@uoc.gr; 4 Department of Biomaterials, Mendeleev University of Chemical Technology of Russia, Miusskaya sq., 9, 125047 Moscow, Russia; yaroslav.mezhuev@gmail.com; 5 Laboratory of Heterochain Polymers, A.N. Nesmeyanov Instituite of Organoelement Compounds, Russian Academy of Sciences, Vavilova St. 28, 119334 Moscow, Russia

**Keywords:** pollution, microplastics, nanoplastics, polypropylene, polyethylene, polystyrene

## Abstract

The increasing awareness of the potential health risks associated with microplastics’ (MPs) and nanoplastics’ (NPs) presence in the environment has led to a significant rise in research focused on these particles over the past few years. This review focuses on the research on MPs’/NPs’ presence and spread, pathways of exposure, toxicological effects on human health and legal framework related to MP/NP challenges. Several research projects have aimed to assess their potential harm to human health, focusing on different systems and organs. After exposure (independent of the pathway), these hazards reach the blood stream and concentrate in different organs. Further, they are responsible for harmful changes, having an immediate effect (pain, inflammation, or hormone imbalance) or lead to a long-term disease (e.g., infertility, chronic obstructive pulmonary disease, or cancer). Toxicological effects have been noticed at high concentrations of MPs, specifically polystyrene, the most widespread typical MP, but only short-term effects have been mostly studied. Significant quantities of consumed MPs have been discovered to have diverse detrimental effects, posing a threat to human welfare. The exact concentrations of microplastics that are inhaled and swallowed and then build up in the human body are still not known. Further investigation is necessary to evaluate the impact of MP/NP contamination at minimal concentrations and for prolonged durations.

## 1. Introduction

Plastic pollution represents the accumulation in the environment of synthetic polymer materials and has become one of the most important threats to human health. The increasing manufacturing of plastic materials and the lack of suitable methods of disposal just overwhelms the ability to deal with them. Even though we recognize the many uses of plastic materials, we need to be aware about the highly increased pollution and the health impact resulting from these materials [1]. The UN report from 2021 (www.unep.org, accessed on 29 September 2024) mentioned that around 400 million tons of plastic are manufactured each year [2,3,4]. Furthermore, the same document is living proof that the highest quantity of plastic was produced after 2000: 9.2 × 10^9^ metric tons of plastic are estimated to have been manufactured between 1950 and 2017, and out of these, more than half has been produced since 2004. Even worse, each year, around 8 million tons of processed plastic are disposed of into the ocean [5]. Unfortunately, all these new materials contain additives (e.g., functional substances, colorants, reinforcement, fillers etc.) that can extend the life of products, with some estimates ranging to at least 400 years being required to break down.

Plastics persistence for extended periods can lead to the contamination of multiple distinct environmental components, such as surface waters, sediments, ground waters, soils, and the atmosphere itself [6,7]. The problem is compounded by their inadequate recycling, which, according to current estimates, only involves approximately 9% of the plastic waste produced, meaning that the remaining 91% stays in the environment [8]. Out of the 30% of the produced plastics remain in use, resulting in the generation of around 6.9 billion tons of primary plastic waste around the world [9]. This plastic waste is made up of 81% polymer resin, 13% polymer fibers, and 32% additives. The environment currently contains various kinds of plastics, the most frequent of which are polyethylene (PE)>, polypropylene (PP)>, polystyrene (PS)>, polyvinylchloride (PVC)>, poly-ethylene terephthalate (PET), which are the main polymer categories also found in freshwater [10].

Microplastics are primarily composed of synthetic polymers derived from petrochemicals, and they often contain a range of additives to enhance their properties. The key components that make up microplastics may be categorized in three major classes: *common polymer types* (PE, PP, PS, PET, PVC, and polyurethane (PU)), *plastic additives* (plasticizers such as phthalates, bisphenol A, flame retardants, stabilizers, colorants, and antioxidants), and *contaminants adsorbed from the environment* (persistent organic pollutants, heavy metals, pesticides, and herbicides). It is important to be aware of the fact that, even though MPs and NPs may have their own harmful effects, human health also impacted by the presence of their secondary components (as above mentioned). Furthermore, sometimes the changes that occur may be even more serious due to this association.

Further, in this review, we focused on the MPs’/NPs’ effects on animal and human health. However, we would like to point out that we must not neglect the harmful effects that some other plastic components may have on different systems and organs. **Bisphenol A** is still a substance under the debate of different agencies all over the world regarding its safety and its activity in the endocrine system. BPA was established to have an estrogenic action and it was demonstrated to induce the cornification of vaginal epithelium after injection into female rats [11]. With its two benzene rings and two (4,4′)-OH substituents, BPA fits within the binding pockets of both estrogen receptor (ER)α and ERβ [12]. Further, BPA is an antagonist of thyroid hormone receptor, interfering with the normal binding of 3,5,3′-triiodothyronine [13,14]. Also, besides its own effect, we have to recognize and to properly document the activity of its metabolites (BPA-glucuronide, BPA-sulfate and 4-methyl-2,4-bis(4-hydroxyphenyl)pent-1-ene—MBP). MBP, even in small concentrations, is 1000 times more biologically active than BPA [15]. 

Other important components with well-noticed effects on human health are the **phthalate esters** (PAEs). These substances are used to make plastics more flexible and durable and are commonly used in PVC products. Even though the general population is more and more exposed to these substances and the effects were identified, it is still necessary to better understand their mechanism of action and to look in more detail at all the important human systems and organs. Chang and colleagues conducted a review of these effects on human health. Their review revealed that multiple epidemiological studies associated the PAE exposure with a decrease in sperm quality in males and the development of symptoms of ADHD in children [16]. 

Similarly to BPA, PAE will interfere with the endocrine system and the functioning of multiple organs. These effects will have, in the end, a negative long-term impact on the success of pregnancy, child growth and development, and reproductive systems in both young children and adolescents [6].

The place where the plastic pollution reaches its peak is definitely the seas and the oceans of the Earth. It is estimated that the plastic litter that reaches the oceans’ water originates from the rivers, carrying more than two million tons of MPs each year [17]. Once these materials arrive in the water, they are exposed to different processes, like UV radiation from the sunlight, air exposure from the wind and, of course, water contact. All these actions determine the degradation of these materials into small particles, which are named microplastics (MPs) if they have a dimension of less than 5 mm or nanoplastics (NPs ) if they are less than 1 µm. Furthermore, these particles break down into even smaller particles that are, these days, spread everywhere: the environment, water, air, and even our human body (blood, lungs, and even feces). These materials persist in the environment for extended periods, hence their widespread presence in ecosystems worldwide [18]. Although the human body is unable to absorb larger-sized microplastics, most plants and organisms living in the soil can easily do so, thus threatening the natural world [19]. Soil is the primary component of all terrestrial ecosystems, serving as a crucial source of vital nutrients for plant development, decomposition of plant matter, and transportation of biomass. Soil plays a crucial role as a natural buffer in the movement of chemical elements and compounds in the atmosphere, hydrosphere, and biota [20]. To improve the effectiveness of how cells absorb nanoparticles and gain a deeper understanding of the physiological process, it is crucial to study the specific mechanisms by which nanoparticles interact with cell membranes.

Taking all these in consideration, the present narrative review aims to show the importance of human acknowledgement about the importance of decreasing the high rate of plastic pollution. This uncontrolled phenomenon has led to the presence of microplastics in the environment, which further negatively impact our health. Also, we explain the path of exposure to these materials and the impact that they have on our health.

## 2. Plastic—Life Process and Degradation

**Figure 1:**
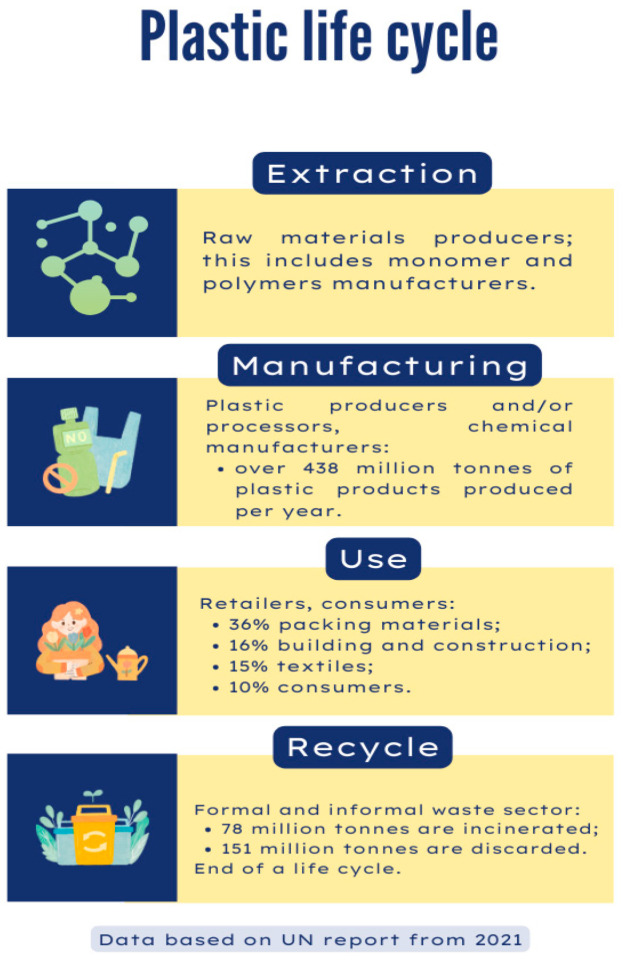
Plastic life cycle.

The life of plastic materials starts with the extraction of raw materials, which are further processed, shaped, and molded into different forms and shapes so that we can have, in the end, the final product that we further use (Figure 1).

**Figure 2:**
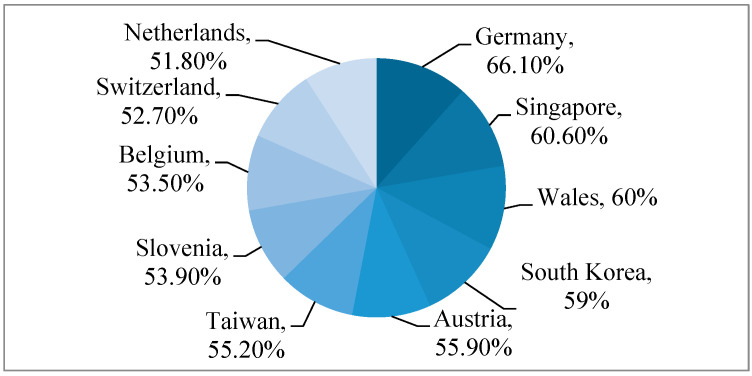
Top 10 countries with the highest recycling rates.

This cycle should end with the collection of the plastic waste that should, after this, enter into an environmentally friendly recycling process. Unfortunately, the occurrence of the re-entry of plastic into a second life cycle is not increasing with the amount of plastic materials being manufactured. Arthur Zuckerman presented in his article from 2020 that more than 33% of the waste in high-income countries ends up in open dumpsites, and the United States of America produces the most trash around the world. On the other hand, Germany has the highest recycling rate of any country in the world, at 66.1% [21]. Based on the statistics we found, the top 10 countries with the highest recycling rates are: Germany, Singapore, Wales, South Korea, Austria, Taiwan, Slovenia, Belgium, Switzerland, and The Netherlands (Figure 2).

**Figure 3:**
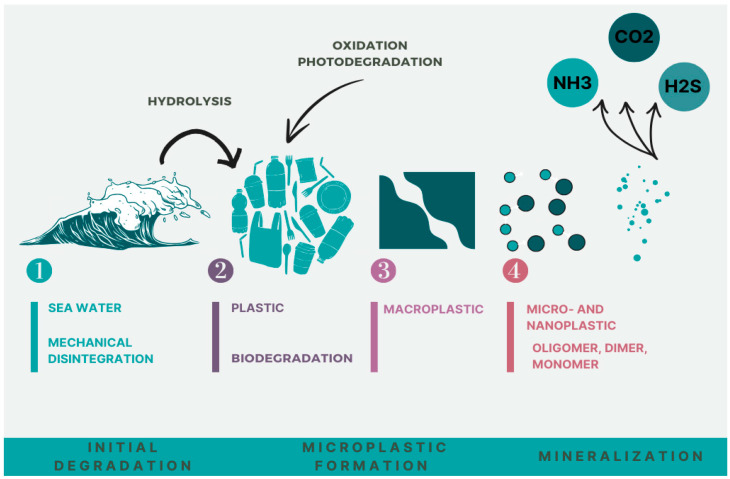
Plastic from water sources degradation pathways.

From a chemical point of view, plastic is a composite material with a matrix, based on synthetic or semi-synthetic polymers. Polymers are molecules of considerable size (macromolecules) with a molecular weight, in most cases, in the range from 104 to 106, built by repeating monomer residues. The plastic degradation processes can be classified as two major types: physical degradation (that refers to structure changes) and chemical degradation (that refers to molecular changes—the cleavage of chemical bonds in the main chain of a macromolecule) [22]. Usually, the second type of degradation involves either hydrolysis or oxidation (Figure 3).

However, both mechanisms may be accelerated by microbes, heat, light, or any combination of those. The results of these processes are the micro- and nanoplastic particles.

Depending on their original shape, degradation processes taking place on the plastic exterior, and their persistence in the surrounding environment, microplastics can be found in multiple forms, such as fibers, fragments, spheres, beads, films, flakes, pellets, and foam [23]. 

## 3. Microplastics—Existence and Over-Abundance

If we look closely, we can identify two major sources of micro- and nanoplastics that can impact the human body: land-based and ocean-based sources. Even though we have different responsible agents for these two roots of exposure, the land-based microplastic hazard is responsible for 80–90% of water-based pollution [24]. Some of the land-based sources of plastics may include different plastic objects (e.g., bottles, food containers, plastic bags, cleaning gloves etc.), care (hand soap, shower gels, face cream, toothpaste, make-up, etc.), and household products (detergents, washing powder etc.), clothing, and plastic incinerators, etc. Moreover, it is important to note that the excessive use of one-time-use facial masks made of plastic polymers (e.g., polyesters and polypropylenes), during the pandemic of coronavirus in 2019 (COVID-19) has significantly increased microplastic waste [25]. Once the micro- and nanoplastics enter the environment, the human body will be further exposed to them using different pathways.

The presence of microplastic pollution in the marine ecosystem was first described in the 1970s, when spherical, disk-shaped, and pellet-like particles were discovered on the surface of the Sargasso Sea, along the shores of New England, and in the surface waters of both the Atlantic and Pacific Oceans [26,27,28]. Taking into account that, on the one hand, sea products (fish, shellfish, and sea salts) are among the primary sources of human food and that, on the other hand, shellfish such as crustaceans and bivalves, as well as edible fish species are frequently subject to microplastic contamination, the marine environment has been extensively researched for microplastic pollution [29]. Microplastics were found in oysters (average MP concentration: 0.33 ± 0.23 n/g), mussels (average MP concentration: 1.21 ± 0.68 n/individual), and Manila clams living on the Korean shoreline [30]. 

The primary source of MP contamination in aquatic settings is plastic waste that is inappropriately disposed of on land. Additionally, though to a smaller extent, certain maritime activities, such as the fishing industry, also contribute to pollution with plastic equipment [31]. Due to certain atmospheric phenomena such as winds, ocean currents, river outflows, and drift, atmospheric and land microplastic particles can be transported to distant regions. This is the basis for the recent inclusion of microplastics among airborne contaminants [32,33,34]. 

In addition to sea waters, however, research has currently been expanded to include the study of wastewater, rivers, and lakes [35,36]. Furthermore, Yanping Tan et al. presented, in their study from 2023, that the major pollution sources of MPs in rivers are wastewater treatment plants, urban and industrial wastewater effluents, atmospheric deposition, and agricultural drainage [37]. 

As far as the presence of microplastics in the soil is concerned, the primary sources are the plastic mulch films used in gardening, compost and municipal solid waste derived from large communities, the resulting bio solids (such as anaerobic processes and sewage sludge), irrigation and the flooding of wastewaters, and atmospheric deposits. However, to build an actual picture of the sources of microplastic pollution of the soil, one should not overlook the illegal disposal of trash and use of plastic-coated fertilizers [38,39]. The annual quantity of plastics accumulated into the soil is considerably higher than the amount released into the oceans [40].

In addition, we present some evidence of the amount of microplastic found as the results of several studies that we were able to find during our research. These data explain that the concentration of MPs found in rivers, seas, and oceans overflows the amount of fish, plankton, and larval fish, approximately ~30:1 (Table 1) [41,42,43]. 

Microplastics are formed as a result of multiple, distinct processes and they are subsequently relocated by various routes to different environmental areas. Thus, they penetrate the food chain and, ultimately, the human body. In this context, it should be noted that the estimated annual maximum human exposure is 6110 microplastic particles. Taking these processes into consideration, the most frequent exposure route for humans is contaminated food (especially fish and other marine dishes). Food contamination can be attributed to factors related to the environment, such as the contamination of water, soils, and air. Additionally, production procedures, namely the use of certain materials during the milk filtration process, for instance, can also contribute to contamination [44,45]. 

Microplastic contamination can also occur as a result of packaging materials, including bottled drinking water, beer, milk, refreshments, and takeout food containers [46]. Furthermore, some studies have been published that focus on the presence of microplastic contamination in the salt for human consumption. These pollutants may be found directly in the finished product or the contamination may occur during manufacturing and processing [47]. Table 2 summarizes the findings related to studies conducted on various brands of sea salt from Europe [48,49,50,51]. 

Keeping in mind that salt is utilized in various food preservation techniques, such as the preservation of fruits, cheese, cereals, and beverages, depending on their chemical properties and affordability, as well as in the cosmetic and the pharmaceutical industries, its microplastic content is an obvious reason for concern. Therefore, the level of salt contamination with plastic micro- and nanoparticles is carefully evaluated by local and worldwide studies. These have shown that the microplastic concentration in salt is significantly influenced by its source. As a result, correlated with the high level of the plastic pollution of sea water, sea salt has the highest degree of contamination, followed by lake salt, rock salt, and well salt, in this order. As concerns the manner in which salt becomes contaminated with plastic particles, it should be noted that bulkier plastic objects are broken down and further turned into microplastics through all three types of environmental degradation (biological, photolytic, and mechanical); thus, salt has been demonstrated to serve as a transporter of microplastics.

Contamination with plastic particles also affects freshwater, which is the primary source of potable water for human usage. It has been revealed that freshwater contains polyethylene (PE) and polypropylene (PP), which make up more than 90% of the MPs in drinking water. Additionally, other materials that are often used for the fabrication of various products, such as polyethylene terephthalate (PET), polystyrene (PS), polyvinyl chloride (PVC), polyester (PES), polyamide (PA), polytetrafluoroethylene (PTFE), and rubber (RY), have also been detected in freshwater. These polymers are also frequently used in the packaging materials employed in the food and cosmetic industries, household goods, and toys [52]. According to the World Health organization, tap water contains approximately 5 particles of microplastics per liter, meaning the intake of a daily dose of 10 microplastic particles for each person drinking the recommended 2 liters of water per day [53,54]. 

Drinking water, sea food, fish, and food preserved using salt, and the use of plastic containers and textiles are not the only sources of plastic penetrating the human body. A further source refers to the technical advancements introduced in industrial milk processing, aiming to improve sanitary conditions and thus decrease potential harms to human health. This, however, has had a significant impact on milk composition, which may introduce microplastics into fluid milk samples. The discharge of microplastics from milk consumption is a significant reason for concern, primarily considering that young children, the most vulnerable age group, are the main demographic category of milk users [55]. In addition, there is evidence that infant feeding bottles, typically composed of polypropylene, have the potential to leak microplastics into milk [56].

Fruits and vegetables have been found to contain microplastics as well (1.36–3.19 μm). The detected levels have been between 52,600 and 307,750 particles/g for fruits and 72,175 and 130,500 particles/g for vegetables. Among the samples studied, apples and carrots were the most contaminated. The projected daily intake of microplastics from these fruits and vegetables ranged from 2.96 × 10^4^ to 1.41 × 10^6^ particles/kg/day [57].

## 4. The Pathways of Exposure to Micro- and Nanoplastics and Their Impact on Human Health

Ingestion, inhalation, and skin absorption are the three main pathways via which microplastics can infiltrate into the human body and cause toxicity [58]. If we look closely, we can assume that the ingestion toxicity associated with MPs and nanoplastics has a higher frequency due to the consumption of contaminated food and water. Moreover, we conducted a research review on different studies that were performed on seafood, ocean organisms, and several types of water.

- *Ingestion* seems to be the main route of exposure, taking into consideration the contamination of different food and water sources. Nanoparticles are prevalent across all the levels of the food chain and have been detected in numerous consumer goods, including salt, sugar, honey, soft drinks, beer, milk, fruit, and water.
- *Inhaled* microplastics can cross the respiratory tract epithelium through diffusion, direct cellular penetration, or active cellular uptake [59]. If compared, it should be noted that the quantity of microplastics inhaled was 3 to 15 times greater than the amount ingested. Therefore, the human intake of MPs through ingestion is minimal in comparison to the overall exposure [60].
- Exposure to microplastics through *direct contact* with the skin is considered a less relevant pathway, but even so, epithelial cells experience oxidative stress when exposed to both micro- and nanoplastics [61,62].

In vitro and in vivo research has outlined the various toxicological profiles of exposure to pollution, specifically microplastics [63,64]. Their toxic effect on organic cellular and molecular components (so far, mainly aquatic organisms, invertebrates, and certain rodents, but humans as well) and the environment in general have been clearly demonstrated. Strong factors influencing microplastics’ harmful effects on the human immune response and cytotoxicity are the exposure time and pollutant size, dose and concentration [65,66,67]. 

The in vivo studies carried out have shown the potential of microplastics to adversely affect organisms [68]. The first to be studied in that respect were aquatic animals, whose organisms have been found to contain cotton and polymers such as nylon, ethylene-propylene, polyethylene, polypropylene, polyethersulphone, and polyester [69,70,71]. 

The source of pollutants’ bio-accretion in aquatic animals lies not only in their specific diet but also in their characteristic, somatic features, such as a water-repelling surface and a higher ratio of surface area to volume. Aquatic mammals are usually exposed to microplastic toxicity by dermal, oral, intraperitoneal, subcutaneous, and intravenous routes [72,73,74]. The route of exposure to microplastics strongly influences their degree of toxicity. Thus, direct contact is the cause of acute toxicity, whereas indirect contact though the food chain leads to chronic organ toxicity [75,76,77]. 

Next, the distribution routes in aquatic organisms were studied, showing that microplastics are distributed mostly to the gastrointestinal tract, the gills, and the muscles, where they also accumulate [78,79]. Due to in vivo research, it has been shown that, in sea animals, microplastics adversely impact the physiology of the gastrointestinal tract, causing, for instance, the dysbiosis of intestinal microbiota, the distortion of healthy metabolism, villi cracking, and enterocyte splitting, among other things. In addition, pollution may lead to the depression of their immune system and detoxification carried out by means of the signaling pathways, involving the c-Jun N-terminal kinases and the extracellular signal-regulated kinases. The level of oxidative stress and the general stress response also becomes higher, as revealed in intestinal tissues by altered glutathione concentrations and elevated superoxide dismutase and catalase amounts [80]. 

Cytotoxicity is also markedly increased and differential gene expression is affected as well. Furthermore, the growth of sea organisms (invertebrates included) is clearly negatively influenced. These finding have been confirmed by the outcomes of in vivo research performed on other organisms such as nematodes, earthworms, arthropods, and rodents. Among these, the exposure of nematodes to microplastics has resulted in the marked down-regulation of gene expression, with harmful effects on gamma-aminobutyric acidergic and cholinergic neurons. On the other hand, in mice, exposure to microplastics alters neurotransmission. Also in mice, microplastic tissue accretion has been shown to be size-dependent, with significant accumulations occurring mainly in the kidneys and intestines [81]. 

The above and other research findings revealing the harmful potential of pollution with microplastics on other organisms have highlighted the imperative for human health of a sound understanding of the human organism response to exposure to microplastic pollution, its sources, and circulation [82]. 

The main sources of microplastic exposure in humans are food contamination (with certain fish and seafood, particularly) and MP-containing water, as well as, though less impacting, skin contact or ingestion via the airways [83,84,85]. Based on the emerging importance of the impact of microplastic pollution on the human body, research has been intensified, seeking mainly to elucidate the toxic potential of microplastics’ harm to humans. Therefore, with epidemiological data lacking, in vitro studies have been performed. These have used human biological samples (e.g., sputum, meconium, feces, colectomy samples, human placenta) and have demonstrated the accretion of microplastics [86,87,88]. 

Such studies have focused on several types of human cells (among which were dermal fibroblasts, pulmonary epithelial, peripheral blood mononuclear, the adenocarcinoma cell line), which were studied in vitro to assess their adverse effects on the body. Thus, microplastic accumulations have been found in the human circulatory system [89,90]. (Although not extensive, existing research data have shown the prevalence of certain predominant microplastic contaminants as follows: high-density polyethylene in human feces and polyurethane, polypropylene, polyethylene, and polystyrene in the placenta and meconium. The presence of microplastic contaminants in colectomy specimens has been very marked.

Further, we discussed the impact of MP/NP toxicity on different organs and systems of the human body, including some analyses that were performed to identify the amount of these harmful agents.

**(a)**  **Gastrointestinal tract**

Since one of the most common pathways of exposure to MP is via contaminated food and water, the gastrointestinal is a system that is often impacted by these agents. MPs can cause physical harm when they are ingested and the physical irritation to the gastrointestinal tract may eventually cause inflammation, resulting in various gastrointestinal symptoms [84]. Due to their small size and non-digestible nature, they accumulate in the gut, potentially causing *mechanical irritation* (the sharp or irregular surfaces of some microplastics could irritate the intestinal lining, leading to local inflammation or abrasions) and *reduced absorption* (MP particles might disrupt the structure and function of intestinal villi, which are responsible for nutrient absorption. Studies in animals have shown that exposure to MPs may reduce nutrient absorption by damaging or blocking these microstructures). Furthermore, the presence of micro- and nanoplastics at this level may disrupt the balance between beneficial and harmful bacteria. This effect may determine various gastrointestinal symptoms: abdominal pain, bloating, and changes in bowel habits [91].

Moreover, the chemical toxicity associated with the ingestion of MPs was identified during different studies. These effects involve the absorption and accumulation of environmental hazards such as additives, heavy metals, or polycyclic aromatic hydrocarbons, since the MPs act as vectors in these cases. These harmful substances can enter the body through the orally ingested microplastics, leading to various gastrointestinal symptoms, such as nausea, vomiting, and abdominal pain [92]. Also, these vectors can help the entrance into the human body of endocrine-disrupting chemicals, such as bisphenol A. Certain chemicals associated with MPs can mimic hormones, potentially leading to disorders in metabolism, reproduction, or other hormonal pathways.

Some studies associated the ingestion of MPs with *oxidative stress and cellular damage*, as a result of extracellular and intracellular processes [93]. Microplastic ingestion has been shown to increase the production of reactive oxygen species (ROS) in gut cells. ROS can damage cellular DNA, proteins, and lipids, which could contribute to chronic diseases such as cancer or metabolic disorders. The toxicity of MPs is size-dependent, and the potential for ROS generation increases with the plastic particles’ size [94]. Increased oxidative stress and cellular damage may eventually lead to programmed cell death (apoptosis) or uncontrolled cell death (necrosis), further damaging the integrity of the gut lining.

A mass spectrometric analysis was performed to evaluate the microplastic content, such as polycarbonate and polyethylene terephthalate, in the fecal samples of adults and children. Contamination with 15 different microplastics was shown in the human feces samples used, indicating ingestion as route of exposure. The most frequent microplastic contaminants were polyamide polyethylene and terephthalate. The amount of polycarbonate content was similar in both the categories of samples, with children being, however, more susceptible to exposure because of their more significant interaction with microplastics in their daily activities (mainly objects used for drinking, feeding, and playing) [95,96]. 

Currently, research on the presence of plastic polymers in feces samples is challenging, mainly because of the lack of standardized methods for their extraction and the difficulty of discriminating inorganic from organic material. In addition, extraction procedures also have to take into account the fragility of the plastic particles, which are sensitive to high temperatures and potent chemical reactions. At present, the extraction techniques involve the digestion of fecal compounds such as lipids, proteins, bacteria, etc., using various enzymes as well as NaOH, HNO_3_, KOH, or H_2_O_2_. For identification, Fenton’s reagents have been suggested, whereas the breakdown can be performed with ethyl alcohol and nitric acid. The residue found on microplastic particles may be cleaned using ethyl alcohol [97]. 

Although a clear causal relationship could not be established, a greater content of microplastics has been found in feces samples from patients with inflammatory bowel disease, and the severity of the disease seems to grow with the presence of these contaminants. The presence of microplastics in saliva, sputum, and lung lavage fluid points to air exposure and inhalation. The air may be contaminated by several routes, one of which may be textile washing, leading to the leakage of microfibers into the water cycle, but also, and less noticeable, by direct release from clothing and other textile materials [98,99,100]. 

As health professionals, we need to also consider the long-term effects that may be associated with the ingestion of MPs and to mitigate against the increasing plastic pollution. While research is ongoing, there are concerns that chronic inflammation and oxidative stress due to micro- and nanoplastic ingestion could increase the risk of gastrointestinal cancers.

The ingestion of microplastics presents a range of possible threats to gastrointestinal health, including physical damage, inflammation, chemical toxicity, and the disruption of the gut microbiota. Although research is still in its early stages, animal models suggest significant risks, and the human health implications are currently under rigorous investigation.

**(b)**  **Cardiovascular system**

As previously described, MPs and NPs are entering the human body using different pathways (ingestion, inhalation, or dermal exposure). Further, these hazards reach the blood stream and are carried throughout the body to different systems and organs [101]. Therefore, it is important to better understand the interaction that takes place between the blood and the MPs/NPs. This action is potentially contributing to cardiovascular diseases (CVDs) through mechanisms such as inflammation, oxidative stress, and endothelial dysfunction. When microplastics enter the bloodstream, they can trigger an immune response similar to other foreign particles. The body’s immune cells may attempt to eliminate or encapsulate the particles, leading to chronic inflammation, which is a known risk factor for cardiovascular disease, contributing to conditions like atherosclerosis. In response to MP/NP exposure, the body can release pro-inflammatory cytokines, such as interleukin-6 (IL-6) and tumor necrosis factor-alpha (TNF-α), which play key roles in vascular inflammation and atherosclerosis development.

Red blood cells (RBCs) are the predominant cellular component of blood, and the biological effects of plastic particles on RBCs can be inferred from studies involving nanoparticles. The exposure of RBCs to polystyrene nanoparticles (PS-NPs) has been shown to induce aggregation and promote adhesion to endothelial cells. These effects are dose-dependent, becoming more pronounced with increasing PS-NP concentrations, particularly in the range of 0.05–0.5 mg/mL, and as particle size decreases [102]. Given that RBC adhesion to vascular endothelial cells is a key factor in the pathogenesis of cardiovascular diseases, nanoparticles may represent a potential risk factor for cardiovascular health.

In a related study, the effects of silica nanoparticles (sNPs) on RBCs were examined. Larger sNPs were found to adsorb onto the RBC surface, causing localized membrane deformation, while smaller sNPs did not elicit similar effects. The hemolytic activity and the internalization rates of the larger sNPs were notably higher, at both 50 and 100 µg/mL concentrations [103]. These findings suggest that the size of plastic particles differentially affects cardiovascular cellular components, highlighting the importance of particle size in determining their biological impact.

Microplastics have been shown to activate platelets and coagulation pathways, increasing the risk of *thrombosis*. This could lead to conditions like deep vein thrombosis or even pulmonary embolism and pulmonary hypertension, all of which are serious cardiovascular events [66]. By interacting with vascular cells and promoting clot formation, MPs could also disrupt normal blood flow, contributing to conditions like *ischemia*.

The most revealing biological sample for contamination with plastic particles is the blood; blood that has not been directly exposed to plastics and was extracted without plastic instruments can show an unadulterated level of contamination. In spite of this lack of direct contact with microplastics, methyl methacrylate, styrene polymers, polyethylene, and polyethylene terephthalate have demonstrated bioavailability in blood circulation.

**(c)**  **Respiratory system**

The presence of microplastics in the respiratory system can result in various harmful effects. Studies have shown that microplastics can trigger inflammation, oxidative stress, and a compromised lung function. Due to their small size, microplastics are capable of penetrating deep into the lungs, reaching the alveoli where gas exchange occurs. This raises concerns regarding long-term health outcomes, including the onset of respiratory diseases and the potential for microplastics to translocate to other organs [104]. 

The data revealed by an analysis for the microplastic content in lung lavage fluid have confirmed the suspected potential of contamination with plastic particles to decrease and damage the lung function. The number of plastic particles identified in these types of samples is significantly greater than those revealed in human feces (21 as compared to 15), and mostly comprised polyurethane particles. 

The size of the MPs is very important when we discuss the effects on the respiratory system. After inhalation, the particles larger than 10 μm will usually collide with the upper airways, but, in the meantime, the particles smaller than 10 μm can reach the bronchioles. Further, the MPs smaller than 2.5 μm are able to penetrate the alveoli. Once these particles enter the respiratory system, it is likely that these hazards will be caught by the fluid lining the lungs [105]. Some studies have shown the presence of plastic in the lungs, proving that MPs and NPs can deposit and build up at this level. Therefore, it was concluded that these particles may be a key factor of various illnesses, such as asthma and pneumoconiosis [105,106].

The presence of contaminant particles in the lungs and the airways can be identified using bronchoalveolar lavage fluid. This involves performing scanning electron microscopy–energy dispersive spectroscopy and Fourier transform infrared spectroscopy on a saline solution previously instilled into the lungs [107].

**(d)**  **Reproductive system**

Unfortunately, the reproductive system is not lacking the harmful effects of micro- and nanoplastic exposure. As they travel through our body, these hazards may produce different changes of the reproductive organs or influence the normal hormonal processes. However, this subject must be explored more and more, as we encounter numerous cases of infertility without sufficient explanations of their cause. Moreover, as modernization increased, the quality of semen decreased. The WHO has published several editions of the Laboratory Manual for the Examination and Processing of Human Semen. In the first edition (published 1980), the normal value for semen concentration was 60 × 10^6^/mL; in the latest volume, the value was reduced to 16 × 10^6^/mL (published 2021) [108].

When assessing sperm quality, the first laboratory test is a semen analysis, but even though we have a global picture of morphological aspects of sperm, this test does not provide information about sperm DNA. The DNA fragmentation index (DFI) represents an important indicator of the integrity of sperm DNA; it was identified that infertile patients have higher values than normal patients [109]. Even though the causes of high values of the DFI may vary, the two main causes are represented by age and exposure to different substances [110]. Furthermore, Amereh and colleagues identified that exposure to NPs with a mean diameter of 38.92 nm for 5 weeks at 1, 3, 6, and 10 mg/kg/day in rats resulted in different degrees of increase in the sperm DFI [111]. 

In their article from 2022, Zhang and colleagues affirmed that for the past 80 years, male semen analysis parameters have shown a significant decline for unknown reasons, speculated to be caused by the exposure to different pollutants. Further, they made a review of the literature and calculated that the minimum human equivalent dose of MPs leading to abnormal male semen quality, which is 0.016 mg/kg/d. All the results that they have found suggest that MPs are one possible cause for semen quality and that they have a major impact on male fertility [112]. 

Multiple studies have been conducted on different mammals to test the response of the reproductive system to different concentrations of MPs and NPs particles. It was identified that polystyrene microplastics (PS-MPs) exert important toxic effects on the reproductive system of male mice, causing a significant decrease in sperm quality and testosterone levels. These particles, being only 0.5, 4, and 10 µm in size entered the three testicular cell types in vitro. A hematoxylin and eosin (H&E) stain showed disorganized spermatogonia, multinucleated gonadotrophic cells in the germinal tubules, inflammation in the testes, and the disruption of the blood–testis barrier.

Another test conducted on mice has shown that PS-MPs can induce reproductive toxicity through oxidative stress and the activation of the p38 MAPK signaling pathway. The results are a decrease in sperm count and motility, increased sperm malformation, and an important reduction of testosterone levels [113]. 

The female reproductive system is also at risk of malformations or processes changes due to MP and NP exposure. Unfortunately, the research must be further developed to properly identify the mechanisms and effects of these hazards to female health. For now, most of the data that we have are based on the analyses conducted on aquatic species and soil fauna and, more recently, also studies in rodents. Malafaia and colleagues conducted a 30-day test on pregnant *Poecilia Reticulata* fish. The animals were exposed to PS-NPs with a size of 23 nm at a concentration of 50 µg/L, which resulted in the accumulation of these particles up until the level of the embryo [114]. These observations are indicating that NPs can pass different important barriers, including the genital tract.

Yang and colleagues presented a comprehensive article related to the impact of MPs and NPs on female reproduction [115]. Studies in rodents have shown that micro- and nanoplastics accumulate in various tissues throughout the body following oral exposure, including the lungs, spleen, liver, kidneys, intestines, brain, and uterus [116]. The cellular uptake and distribution of nanoparticles are highly influenced by factors such as particle size, shape, stiffness, and surface area [117]. In rodent studies, oral exposure over 20–44 days to polystyrene micro- and nanoplastic particles of different sizes (ranging from 50 nm to 10 µm) and at concentrations from 0.015 to 30 mg/kg/day demonstrated the accumulation of these hazards in testicular and ovarian tissues [118,119]. 

Another study from 2023 conducted by Paola Pontecorvi and colleagues concluded that acute exposure to a high concentration of micro- and nanoplastic particles induced cell toxicity in vaginal keratinocytes after effective cellular uptake, as their viability and apoptosis data suggest, along with their transmission electron microscopy (TEM) observations. The presence of these hazards determine changes to the expression of junctional and adhesion proteins, as well as the organization of the actin cortex, affecting the expression of genes associated with oxidative stress signaling pathways and miRNAs related to epithelial barrier function. The beneficial thing was that once the exposure was stopped, the cells were able to recover from the adverse effects within few days. However, in all instances, micro- and nanoplastic exposure led to a persistent alteration in the expression of DNA methyltransferase and DNA demethylase, which could influence epigenetic regulation, potentially accelerating cell aging, promoting inflammation, or leading to malignant transformation [120]. 

The fresh discovery of plastic particles in a human placenta using Raman microspectroscopy has been a source of concern due to the potential of the plastics to affect in utero development [121]. A careful sample examination in a strictly controlled environment to avoid the cross-contamination of samples identified 12 types of plastic particles.

Even though we are still lacking in studies related to MPs and NPs and their influence on the reproductive female system, Luigi Montano and his colleagues conducted the first study of the MPs’ presence in ovarian follicular fluid. The samples of ovarian follicular fluid of 18 women that were under a reproductive treatment were analyzed using a patented method. MPs of dimensions < 10 µm were detected in 14 out of 18 samples of follicular fluid, with an average of 2191 p/mL (0–7181 p/mL) and with a mean diameter of the MPs of 4.48 µm (3.18–5.54 µm). A correlation was found with the FSH, but no other with the fertilization outcomes, miscarriages, or live birth [122].

**(e)**  **Hair and skin**

One study has indicated the superiority of hair and skin samples in comparison to saliva with respect to indicating microplastic contamination in humans [123,124].

Microplastics (MPs) and nanoplastics (NPs) are increasingly concerning in dermatology due to their pervasive occurrence in cosmetic products and the environment. A study indicated that nanoparticles up to 200 nm might penetrate the skin’s furrows, lipid channels, and vellus hair follicles. Nanoparticles can also aggregate on the viable epidermis just under the stratum corneum and even within cells [125]. 

Experimental findings in zebrafish indicate that NPs can induce apoptosis in up to 54% of cell populations by dermal diffusion. The concentration of MPs/NPs was detected during zebrafish embryogenesis prior to mouth development, indicating passive diffusion via the skin [126]. 

Consequently, this evidence may indicate that skin, oral cavity, and scalp hair function as significant passive sensors for microplastics. The capacity to target nanoparticles to hair follicles is being utilized to develop nanoparticle-based cosmeceuticals, transdermal medication delivery systems, and immunization techniques [127]. 

## 5. Microplastics in Urban Zones—Current Challenges

Reports have thoroughly documented the presence of plastic contamination in natural aquatic ecosystems spanning from tropical to Arctic regions. Nevertheless, the extent of microplastic fragments in drinkable water sources, such as water obtained from centralized distribution infrastructure, water that is bottled (such as spring water), the water from wells (groundwater used for diverse uses), and other refreshments consumed by people, is not adequately documented in comparison to natural water bodies. The widespread presence of microplastics in various forms of water, including groundwater, surface water, and wastewater, has prompted inquiries about the potential contamination of water for human consumption. Although drinking water is a significant component of the everyday diet, as it provides necessary minerals and trace vitamins and nutrients, there is not a lot of information regarding the contamination of potable water by microplastics [10]. Moreover, the exposome reflects the impact that environmental factors will influence human health [128]. 

Contemporary civilization is unavoidably reliant on polymers from plastic to such an extent that, up to now, plastics are the foremost sources of pollution on a global scale. MPs were found in numerous ecological matrix structures, especially drinking water sources designated for consumption by people, such as rivers, lakes, and groundwater [129]. 

The process of microplastic fragmentation can be linked to several factors, such as stress from mechanical contact, UV radiation, poor material quality, aging, and atmospheric accumulation. In addition to these, MPs serve as a repository for a variety of chemicals and have the ability to absorb additional intricate substances from their environment. This exacerbates the complexity of microplastic pollution and renders their precise detection in a singular method more challenging. Furthermore, a prevalent habit within communities involves the frequent and prolonged use of plastic water bottles and food containers, which leads to the release of microplastics and poses possible health risks to consumers [130]. 

In urban areas, the lifestyle has changed a lot in recent years, as well as the diet. A series of plastic products are used, especially in the food sector, such as bottles for drinking water or soft drinks, baby feeders, plastic tableware, and food containers. Further in our article we summarize the impact that MPs from different sources impact human and animal health:

- Microplastics in water containers;
- Microplastics in water from pipes;
- Microplastics in food packaging.

Microplastics in water containers

The consumption of bottled water has experienced a significant explosion, over 6000 million gallons per year, in recent decades [131]. Bottled water is commonly used in several places worldwide due to its high level of cleanliness, natural flavor, and convenient portability [132].

Although significant measures have been taken to guarantee the safety of bottled water, it is important to acknowledge the potential for the accumulation of microplastic during different stages of production and consumption, which cannot be disregarded [133]. The container’s body and top are believed to be the likely sources of microplastic contamination in bottled drinking water. Researchers proposed that reusing plastic containers for water exhibited a higher level of microplastics compared to one-time use and recently manufactured containers [134]. UV radiation from sunlight may additionally increase the absorption potential of plastic particles and their agents during the process of shipping and storing them. The researchers discovered that the water from the bottle exposed to sunshine has 326.2 microplastic particles per liter, whereas the bottled water not exposed to light involves a total of 180.7 microplastic particles per liter [135]. Light from the sun or ultraviolet (UV) radiation has the capacity to extract microplastics from beverage containers and lets them to spread into the beverage. The principal routes of plastic contamination originating from beverage containers: leaching, repeated washing, squeezing, thermos-degradation, and industrial washing.

Bottled drinking water is packed utilizing glass as well as plastic constituents. Plastic containers are made up of PC (polycarbonate), PET (polyethylene terephthalate), and HDPE (high-density polyethylene). A total of 259 containers of mineral water were gathered from 11 zones and the researchers discovered that the polymer that was most plentiful in their study was polypropylene, and it was characterized by a fibrous structure. The mean concentration of microplastics among them ranged from 0 to 10,000 pieces per liter, with a mean of 350 units per liter [136]. Another study examined 11 prominent Iranian-market packaged mineral water brands. Contamination with microplastics was found in 9 out of 11 brands, with the majority being in the form of fragments (93%). The mean amount of microplastics was 8.5 ± 10.2 pieces per liter. The predominant plastics identified in this research were polyethylene terephthalate (PET), polystyrene (PS), and polypropylene (PP) [137].

Microplastics in water from pipes

The existence of waste plastic particles in water from the tap is caused by pollution in the system that distributes water, originating from either processing operations or from the pipes directly, or by textile water pollution.

Utilized granular activated carbon (GAC) is used to filter tiny particles of microplastic and has a suggested efficiency range of 56–61%. A recent study analyzed the plastic particle quantities in water supply systems and concluded that the release of microplastics from pipes is a significant factor that should not be disregarded [138]. The most identified routes of plastic contamination originating from tap water: textile sources, water treatment plants, pipelines, and atmospheric sources.

Microplastics in food packaging

The quantity of microplastics found in alimentary packaging is linked to the process of production and the sort of plastic used. The exaggerated utilization of plastic-based packaging for food, baby feeders, throwaway glasses, and glasses presents the real danger of plastic particles leaching into aliments [139]. This problem could potentially be exacerbated, since the COVID-19 epidemic amplified the utilization of packaging made from plastic for the transportation of food. Additionally, a separate study indicated that a significant quantity of microplastics, numbering in the millions, may originate from plastic containers, single-use cups, or clear and flexible food containers when they come into contact with boiling water and food that is heated [140]. The presence of hot water or hot meals may contribute to the movement of microplastics into the meal [141]. Supplementary research has demonstrated that bisphenol A (BPA), fluorescent components, and other substances that disrupt the endocrine system can also be released from plastic packaging, coupled with microplastics, when exposed to high temperatures [142]. 

The presence of oil in fried food and the incorrect utilization of plastic wrapping, such as using it for micro-wave cooking or freezing, may result in the release of microplastics into the meal, hence elevating the potential for contamination by humans and associated risks [143].

Nevertheless, the comparatively smaller quantity of microplastics in tap water as compared to freshwater sources suggests an important level of elimination of MPs in drinking water treatment facilities.

## 6. Legal Framework Related to Microplastic Challenges

Since the issues related to microplastic pollution are growing larger and larger, and are impacting all types of environments, all over the earth, legal measures need to be in place in order to protect and prevent the spread of these contaminants. Their persistence and pervasive nature lead to harmful impacts on wildlife, marine ecosystems, and potentially on human health through the food chain. Addressing this issue requires comprehensive legal frameworks that regulate the production, use, and disposal of plastics, specifically targeting microplastic pollution. Such regulations are crucial for mitigating environmental contamination, protecting biodiversity, and ensuring the safety and well-being of current and future generations. Taking this into consideration, several legal bodies already implemented different measures to prevent plastic pollution at the international and national level. Further, we presented a short overview for each important legal body and their actions against plastic pollution.

**(a)**  **United Nations Environment Programme (UNEP)**

The United Nations Environment Programme plays a crucial role in addressing the global issue of microplastic pollution through various initiatives, partnerships, and publications. We mention below some of the most important actions conducted by the UNEP related to microplastics:

*Global Partnership on Marine Litter* (GPML): launched in 2012, the GPML is a voluntary multi-stakeholder partnership that aims to protect the global marine environment, human well-being, and animal welfare by addressing the global problem of marine litter, including microplastics. The GPML facilitates the coordination and implementation of activities to prevent and reduce marine litter (UN Environment Programme).

*Clean Seas Campaign*: this campaign (launched in 2017) aims to engage governments, the general public, and the private sector in the fight against marine plastic pollution. The campaign encourages commitments from various stakeholders to reduce the production and consumption of single-use plastics and to address microplastic pollution (Cleanseas).

*Assessment Reports and Research*: UNEP conducts and publishes comprehensive assessments on the state of plastic pollution, which includes also the challenge of microplastics disposed in the environment. These reports provide scientific evidence on the sources, pathways, and impacts of microplastics, guiding policy development and implementation (UN Environment Programme, Marine plastic debris and microplastics) [144,145].

Furthermore, the UNEP has established different important collaborations to grow the awareness related to plastic and microplastic pollution, to enhance the research and implement different projects that aim to reduce the pollution. Moreover, the UNEP works with governments to develop and enforce regulations to control microplastic pollution.

**(b)**  **European Union (EU)**

The EU is an important and active body that conducts different actions and projects to fight against plastic pollution and improve the quality of our environment. For the past few years, The EU have implemented different measures to help reduce the plastic pollution.

*Directive (EU) 2019/904* [37]. on the reduction of the impact of certain plastic products on the environment (*Single-Use Plastics Directive*) (Directive EU 2019/904): this directive targets the reduction of single-use plastics, which are major contributors to marine litter and microplastics. It bans specific single-use plastic products for which alternatives are readily available (for example: cutlery, plates, straws, etc.). It also mandates Member States to achieve a reduction in the consumption of single-use plastic products and to implement extended producer-responsibility schemes to cover the costs of waste management and clean-up.

*REACH Regulation:* the EU’s REACH (ECHA) (Registration, Evaluation, Authorisation, and Restriction of Chemicals) regulation includes measures to control the use of intentionally added microplastics in products. In 2019, the European Chemicals Agency (ECHA) proposed restrictions on the microplastics added to products (for example: cosmetics, detergents, and agricultural products). These restrictions aim to prevent microplastics from entering the environment by requiring that alternatives are used or that products are reformulated to eliminate microplastic content.

*Marine Strategy Framework Directive* (MSFD) (Directive 2008/56/EC): this strategy aims to achieve a Good Environmental Status (GES) of the EU’s marine waters and protect the resource base upon which marine-related economic and social activities depend. Furthermore, Member States are required to monitor and assess the impact of microplastics on the marine environment [146]. 

*Circular Economy Action Plan* (COM/220/98)*:* as part of the European Green Deal, the Circular Economy Action Plan aims to reduce waste and ensure that resources are kept in use for as long as possible. It includes measures to prevent plastic waste, promote the use of recycled plastics, and reduce the leakage of plastics into the environment. The European strategy of circular economy was adopted in 2018.

Furthermore, EU implements different research and innovation programs (for example: *Horizon 2000* and *Horizon Europe*) as well as education and awareness campaigns. Through these comprehensive measures and on-going initiatives, the EU aims to significantly reduce microplastic pollution, safeguard marine environments, and promote a sustainable circular economy.

**(c)**  **European Food Safety Authority (EFSA)**

The organization has been actively researching and evaluating the potential risks of microplastic pollution, particularly concerning food safety. The EFSA conducts risk assessments to understand the potential health impacts of micro- and nanoplastics in food. The focal point is seafood, since it is considered to be the major dietary source that is exposed to plastic pollution. The assessments concentrate on toxicity evaluation, the level of exposure, and the health risks associated with the intake of microplastics. In 2016, the EFSA published their scientific opinion over the presence of plastics (micro- and nanoplastics) in food, especially in seafood.

Through their research, the EFSA established two major potential health risks associated with in the ingestion of microplastics: chemical contaminants (micro- and nanoplastics can absorb and transport different harmful chemical substances such as heavy metals, plastic additives, and persistent organic pollutants) and physical effects (the presence of these chemicals in the gastrointestinal tract could cause inflammatory diseases or other severe adverse effects, although more research is need to strongly confirm these effects in humans) [147,148]. 

Unfortunately, the EFSA also identified some major gaps in the current knowledge over the health impact of micro- and nanoplastics. For example, it does still not fully understand the toxicological impact on the human body, the behavior in the gastrointestinal tract, and especially the long-term effects. Therefore, we should prioritize research and conduct multiple studies on the occurrence of microplastics in various foods and also study closely their toxicokinetics as well as the health impact of these contaminants.

However, the EFSA advises caution but does not recommend specific dietary changes due to the uncertainties surrounding the health risks of microplastic ingestion. The authority continues to review new scientific evidence and update its guidance as more information becomes available.

**(d)**  **U.S. Food and Drug Administration (FDA)**

The U.S. Authority plays a major role in addressing microplastic pollution, particularly focusing on the safety of food, beverages, water, and cosmetics that may contain microplastics [149,150]. The FDA collaborates with other federal agencies, academic institutions, and international organizations to conduct and support research on micro- and nanoplastics. This research aims to better understand the sources, prevalence, and health effects of microplastics in food and cosmetics, and also to gain knowledge on how to decrease the harmful potential that these contaminants have.

Furthermore, as with other important international organizations, the FDA is conducting periodic risk assessments in order to evaluate the impact of microplastics on the human body, especially after contaminated food intake. These reports talk about different factors such as the concentration of contaminants in food and cosmetics, their chemical structure and composition, and the risk associated with these contaminants. In the future, the FDA is planning to develop and implement more specific regulations or guidelines to increase the safety of products that are under their jurisdiction. One good example is represented by the guidance issued to food and cosmetic industries on the best practices for minimizing microplastic contamination. This includes recommendations for ingredient sourcing, manufacturing processes, and product testing.

Related to the FDA’s findings and concerns, the Authority is continuously reviewing the health risks and the exposure level of the population to microplastics. While studies have shown that microplastics can carry harmful chemicals and pathogens, the extent of their impact on human health through food and cosmetic exposure remains uncertain. The factors taken in consideration are the size, shape, and chemical composition of microplastics, as well as the likelihood of human ingestion or absorption.

The FDA acknowledges the need for more research to determine the potential toxicological effects of microplastics and to develop standardized methods for their detection and analysis.

**(e)**  **U.S. Environmental Protection Agency (EPA)**

The EPA has recognized microplastic pollution as a significant environmental issue and has been actively involved in research, monitoring, and developing strategies to address it. Taking this into consideration, the Agency conducts and supports research to understand the sources, fate, and, most importantly, the effects of microplastics in the environment and human body. Their focus is to understand how microplastics are transported and move through water, soil, and air, as well as their impact on different ecosystems and human health. The Agency identified various sources related to microplastic pollution, including plastic debris breakdown, microbeads in personal care products, synthetic fibers from clothing, and industrial processes [151]. 

The EPA regulates pollutants under various laws that can apply to microplastic pollution, such as the *Clean Water Act* (CWA) and the *Resource Conservation and Recovery Act* (RCRA). Furthermore, the EPA’s *National Pollutant Discharge Elimination System* (NPDES) permits help control the discharge of pollutants, including plastics, into U.S. waters [152].

Also, during the past few years, the EPA has issued some important reports and initiated programs, such as the *EPA research on microplastics* (the research focuses on understanding the extent of microplastic pollution, its sources, and impacts), the *Microbead-Free Waters Act of 2015,* and the *EPA’s Trash-Free Waters Program* (EPA Trash Free Waters) (this program aims to reduce aquatic trash, including microplastics, through partnerships, policy development, and community engagement) [151].

**(f)**  **Organisation for Economic Co-operation and Development (OECD)**

Another important legal body fighting against microplastic pollution is the OECD, which addresses environmental issues by promoting policies that improve the economic and social well-being of people around the world. The organization conducts extensive research on plastic pollution (including micro- and nanoplastics) and publishes reports that provide insights into the sources, pathways, and impacts of microplastics on the environment and human health. Using these reports, policy recommendations to member countries are made. Furthermore, these documents serve as a basis for developing strategies to combat microplastic pollution.

The organization studies the pathways through which microplastics enter and move through the environment, including their presence in marine and freshwater systems, soil, and the atmosphere. Furthermore, research supported by the OECD examines the ecological effects of microplastics on aquatic and terrestrial ecosystems, which include assessing the impact on biodiversity, food webs, and ecosystem services. The OECD also investigates the potential human health impacts, particularly of the ingestion of microplastics via food and water.

The OECD also focuses on the economic impact of microplastic pollution, including the costs associated with clean-up, the loss of ecosystem services, and public health expenditures. The organization evaluates the effectiveness of existing policies and regulatory frameworks in member countries and provides recommendations for improvement.

As with the other prior mentioned organizations, the OECD collaborates with international organizations, such as the United Nations Environment Programme (UNEP) and the European Union (EU), to address microplastic pollution on a global scale. This collaboration involves sharing research findings, harmonizing methodologies, and developing coordinated strategies. The OECD engages with a wide range of stakeholders, including governments, industry, non-governmental organizations, and academia, to develop and implement effective strategies for reducing microplastic pollution. Public–private partnerships are encouraged to drive innovation and investment in sustainable solutions [153].

Last, but not least, the OECD has published several important reports, such as the OECD *Report on Microplastics in Water* (OECD 2021) (an overview of the sources, pathways, and impacts of microplastics in water bodies) and the *Policy Highlights on Plastic Waste and Recycling* (OECD 2018) (insights into the challenges and opportunities associated with managing plastic waste; this includes recommendations for reducing microplastic pollution through improved waste management and recycling practices). Moreover, The OECD promotes the concept of *Extended Producer Responsibility* (OECD 2024) (EPR), which holds producers accountable for the end-of-life management of their products. EPR schemes can help reduce plastic waste and microplastic pollution by encouraging more sustainable product design and production practices [154,155]. 

The OECD plays a vital role in addressing microplastic pollution by conducting research, providing policy recommendations, and fostering international collaboration. By promoting sustainable practices and encouraging innovation, the OECD aims to mitigate the environmental and health impacts of microplastics and contribute to global efforts to reduce plastic pollution.

The concerted efforts of global organizations and legal frameworks highlight the critical importance of addressing plastic pollution, particularly microplastics, due to their pervasive environmental and health impacts. The United Nations Environment Programme (UNEP) drives international cooperation and policy making, emphasizing a global approach to combating plastic pollution. The European Union (EU) implements comprehensive regulations such as the Single-Use Plastics Directive and the REACH Regulation to reduce plastic waste and promote a circular economy. The U.S. Environmental Protection Agency (EPA) and the Food and Drug Administration (FDA) focus on research, monitoring, and regulating the contaminants in food, water, and cosmetics, while the Organisation for Economic Co-operation and Development (OECD) provides extensive research and policy recommendations to member countries. Collectively, these organizations and legal frameworks aim to mitigate the environmental and health risks of plastic pollution through research, regulation, innovation, and international collaboration. Their actions underscore the urgent need for sustainable solutions and effective management practices to safeguard ecosystems and public health.

## 7. Conclusions

The build-up of plastic microparticles in the environment causes ecological damage and is one of the main results of plastic pollution. Furthermore, according to some studies, the consumption of food and water from various sources is considered a significant contributor to the intake of microplastic particles. High amounts of ingested micro- and nanoplastics have been found to have various harmful consequences, representing a hazard to human well-being. The present review aimed to put together multiple study results, concluding that the exposure to these hazards will determine their accumulation in the human body. Further, these particles reach the blood stream, that will transport them to different systems and organs. At each level, MPs and NPs will determine various harmful effects: gut microbiome disruption (which may result in inflammation, leaky gut syndrome, and digestive disorders), lung inflammation, and respiratory diseases (e.g., chronic obstructive pulmonary disease or even lung cancer, especially via occupational exposure), atherosclerosis, stroke, hormonal imbalance, etc. Furthermore, recent studies show that the presence of MPs and NPs may affect sperm quality and may accumulate in testicles and ovaries, leading to harmful changes in the reproductive system. The precise concentrations of microplastics that are inhaled and swallowed and subsequently accumulate within the human body still remain unknown. Insufficient data currently exist about the direct impact of plastic particles on the well-being of people. Further studies should give priority to investigating the specific impacts of microplastics on human health, at concentrations that accurately represent real-world environmental exposure. Also, new viewpoints may be taken in consideration such as the fact that these particles serve as a vector for other hazards, antibiotic resistance, epigenetic changes, impacts on fetal development, neurotoxicity, and brain health, etc. Moreover, we should not neglect the development of new ways of reducing plastic pollution, so that we can decrease the interaction between MPs/NPs and animals, plants, and humans.

## Figures and Tables

**Table 1:** Microplastic contamination in different waters and their biota.

	MP Conc. in Water (MP × L^−1^)	MP Conc. in Sediments [MP × (kg Dry Mass)^−1^]	MP Conc. in Biota ^b^		
Watershed	Surface	Beach ^a^	Fish	Birds	Frogs
Lauretian Great Lakes, USA and Canada					
Lake Erie and tributeries	<0.001–0.032	50–391	70%	1.8–9.8	
Lake Ontario and tributaries	0.002–1.5	20–4270	50%	1.8–9.8	
Lake Michigan and tributeries	<0.001–0.007		0.19–1		
Milwaukee River	0.002–0.017		4.5–6.5		
Canada (Baynes Sound, Vancouver Island)	0.69 MP/L (1 L samples) and 0.12 MP/L (10 L samples)				
Yangtze River Basin, China					
Three Gorges Reservoir	4.7–12.6				
Yangtze River Delta inland waters	0.5–21.5				0.17–3.51
Lake Taihu	0.53–25.8		0.2–17.2		
Lake Poyang	0.24–34		0–18		
Other					
Rhine River, Europe	0.005–0.022		0.2–1.0		
Rize inland waters, Turkey	1.0–13.0				124–489 × g^−1^
Lake Victoria, Tanzania and Uganda	0.02–2.19		20%		
Melborne inland waters, Australia	0.03–1.7		0.7		

a—beach concentrations include samples taken in areas that are never, or only temporarily, submerged. b—the concentration of MP is reported as numbers of particles per individual; where data are not available, the presence of MP is reported as either the proportion of animals contaminated (%) or as the number of particles per gram of tissue (g).

**Table 2:** Strength and size of different types of MPs found in various sea salt brands from Europe.

Countries	Brand (Number of Brands Examinated)	MP Conc. (Particles × kg^−1^)	MP Type	MP Size (µm)
Europe				
France (Atlantic Ocean)	6	0–2	PE, PET PP	160–980
Portugal	3	0–10	PET, PP	160–980
Spain (Atlantic Ocean)	4 (fine salt)	50–150	PE, PET PP	30–3500
	3 (coarse salt)	95–140		
Spain (Mediterranean Sea)	7 (fine salt)	80–280	PE, PET PP	30–3500
	2 (coarse salt)	60–65		
UK	1	120	PP, PE, PVC	100–2000
Bulgaria	1	10	Nylon, PE, PP, PVC	100–4000
Croatia	5 (fine salt)	13,500–19,800	PE, PP	15–4628
	1	800	Nylon, PE, PET, PP	100–5000
Italy	6 (fine salt)	22–594	PE, PP	4–2100
	2	5–50	Nylon, PE, PET, PP	100–5000

PE—polyethylene; PP—polypropylene;; PVC—poly(vinyl chloride); PET—polyethylene terephthalate.

## Data Availability

The data that support the findings of this study are available from the corresponding author upon reasonable request.
